# Author’s reply to: cancer risks of firefighters: a systematic review and meta‑analysis of secular trends and region‑specific differences

**DOI:** 10.1007/s00420-020-01625-3

**Published:** 2021-01-21

**Authors:** Swaantje Casjens, Thomas Brüning, Dirk Taeger

**Affiliations:** Institute for Prevention and Occupational Medicine of the German Social Accident Insurance, Institute of the Ruhr University Bochum (IPA), Bürkle-de-la-Camp-Platz 1, 44789 Bochum, Germany

Dear Editor,

We thank Dr. Jalilian and colleagues for their comments (Jalilian et al. 2020) on our article (Casjens et al. 2020) and we appreciate to discuss the issues raised.

The authors argue that inconsistent findings across different types of cancers might be caused by the selection of random time intervals. As we mentioned in our paper, polymers were used in large quantities from the 1950s onwards (Guidotti and Clough 1992). We also explained that firefighters’ PPE changed dramatically over time, with the use of modern self-contained breathing apparatus (SCBA) being one of the standard equipment used by firefighters from the 1970s until today (Misner et al. 1987). Due to these facts, we have divided the studies into the reported three different time periods. We agree with Jalilian et al. that meta-regression is a powerful tool in meta-analysis. However, conceptually meta-regression does not differ a lot from a subgroup analysis, especially when a meta-regression with categorical predictors is performed. For this reason, a minimum number of studies is also required for meta-regression, both in modelling with categorical and continuous predictors, to assure reliable conclusions. Meta-regression is especially prone to false-positive results if there are few studies and heterogeneity is present, a situation which we often encounter here (Higgins and Thompson 2004).

In our opinion, the grouping of different countries into regions is permissible, especially since there are a paucity or even no studies in many countries. Since there are certainly always differences between countries this allocation can give only indications. In particular, we consider the combination of the USA with Canada to be less critical than the combination of Asian and Oceanic countries. In fact, we have also carried out sub-analyses, such as the analysis concerning malignant melanoma of the skin. For this type of cancer, we also found higher incidence rates in studies from Australia and New Zealand (mSIR = 1.43, 95% CI 1.27–1.58) compared to other study countries (mSIR = 1.05, 95% CI 0.62–1.49). We suspected that the cause was exposure to strong sunlight rather than occupational exposure as a firefighter.

In our paper, we have already mentioned the small number of eligible studies, especially after stratification with regard to employment period and region, is a limitation. Furthermore, we addressed the heterogeneity between the studies and stated that changes in the observed risks cannot be ruled out after the publication of new studies.

We agree with Jalilian et al. that the quality of studies on cancer in firefighters is very heterogeneous. However, the use of the Newcastle–Ottawa Scale (NOS) to assess the quality of the studies is questionable because it may lead to arbitrary results (Stang 2010). We are surprised that this measure has been proposed again, although the authors previously agreed with us that the NOS is not sensitive enough to differentiate well between studies on cancer in firefighters (Jalilian et al. 2019).
